# Quantitative proteomics of nutrient limitation in the hydrogenotrophic methanogen *Methanococcus maripaludis*

**DOI:** 10.1186/1471-2180-9-149

**Published:** 2009-07-23

**Authors:** Qiangwei Xia, Tiansong Wang, Erik L Hendrickson, Thomas J Lie, Murray Hackett, John A Leigh

**Affiliations:** 1 Department of Chemical Engineering, Box 355014, University of Washington, Seattle, WA 98195, USA; 2 Department of Microbiology, Box 357242, University of Washington, Seattle, WA 98195, USA; 3 University of Wisconsin-Madison, Department of Chemistry, Madison, WI 53706, USA

## Abstract

**Background:**

Methanogenic Archaea play key metabolic roles in anaerobic ecosystems, where they use H_2 _and other substrates to produce methane. *Methanococcus maripaludis *is a model for studies of the global response to nutrient limitations.

**Results:**

We used high-coverage quantitative proteomics to determine the response of *M. maripaludis *to growth-limiting levels of H_2_, nitrogen, and phosphate. Six to ten percent of the proteome changed significantly with each nutrient limitation. H_2 _limitation increased the abundance of a wide variety of proteins involved in methanogenesis. However, one protein involved in methanogenesis decreased: a low-affinity [Fe] hydrogenase, which may dominate over a higher-affinity mechanism when H_2 _is abundant. Nitrogen limitation increased known nitrogen assimilation proteins. In addition, the increased abundance of molybdate transport proteins suggested they function for nitrogen fixation. An apparent regulon governed by the euryarchaeal nitrogen regulator NrpR is discussed. Phosphate limitation increased the abundance of three different sets of proteins, suggesting that all three function in phosphate transport.

**Conclusion:**

The global proteomic response of *M. maripaludis *to each nutrient limitation suggests a wider response than previously appreciated. The results give new insight into the function of several proteins, as well as providing information that should contribute to the formulation of a regulatory network model.

## Background

Methanogenic Archaea (methanogens) occupy a distinct position in phylogeny, ecology, and physiology. Occupying much of the phylum Euryarchaeota, and widespread in anaerobic environments, these organisms produce methane as the product of energy-generating metabolism [1]. Hydrogenotrophic methanogens specialize in the use of H_2 _as electron donor to reduce CO_2 _to methane. The pathways of methanogenesis are well characterized and the proteins that catalyze steps in the pathways are known.

We are engaged in a long-term effort to understand regulatory networks in hydrogenotrophic methanogens. Our studies focus on *Methanococcus maripaludis*, a model species with tractable laboratory growth characteristics and facile genetic tools. Previous studies in *M. maripaludis *have begun to reveal both mechanisms of regulation and global patterns of gene expression. Many of these studies have concentrated on the effects of certain nutrient limitations. For example, at the mechanistic level, transcription of genes encoding nitrogen assimilation functions is governed by a repressor, NrpR, which is found in many Euryarchaeota as well as certain Bacteria and mediates the organism's response to nitrogen limitation [2-4]. However, a global assessment of the response to nitrogen limitation has not previously been conducted in hydrogenotrophic methanogens. At the global level, our previous studies have addressed the effects on the transcriptome of H_2_-limitation, phosphate-limitation, and leucine-limitation [5,6]. The effects of these nutrient limitations at the proteome level have not previously been studied. We have also determined the effects on the transcriptome and proteome of a mutation in a hydrogenase gene [7,8].

Here we focus on the effects of certain nutrient limitations on the proteome of *M. maripaludis*. We report on the effect of limiting H_2_, the electron donor of hydrogenotrophic methanogenesis, and of limiting basic nutrients of biosynthesis: nitrogen and phosphate. Key aspects of our approach include the use of continuous culture for maintaining defined nutrient conditions [9], and exhaustive sampling of the proteome to obtain statistically reliable quantitative information (Xia Q, Wang T, Hendrickson EL, Leigh JA, Hackett M, manuscript in preparation).

## Results and discussion

### Approach

We used chemostats to grow *M. maripaludis *under three different nutrient limitations (nutrient-controlled growth) [9]. Thus, growth was limited by the supply of H_2_, ammonia, or phosphate to grow cultures that were H_2_-limited, nitrogen-limited, or phosphate-limited, respectively. The dilution rate (and hence growth rate) was held constant, and the limiting nutrient was provided at a level that limited cell density to a similar value in each case. As before [5,6], this approach allowed us to obtain a rigorous assessment of the effect of each nutrient limitation without complications arising from variations in growth rate or cell density.

**Figure 1:**
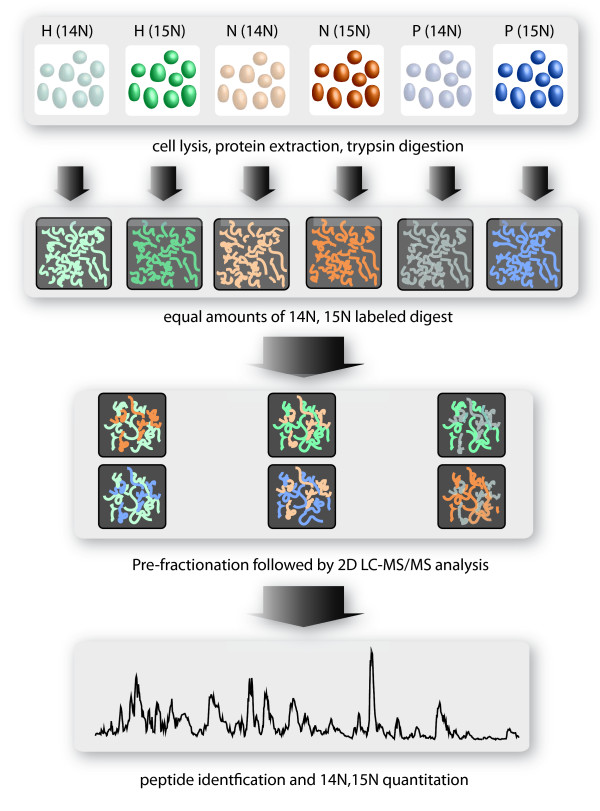
**Experimental design, sample handling and raw data acquisition**. The bottom panel is a representation of a single reversed-phase elution during the final stage of the 2-D HPLC tandem MS analysis, total signal (reconstructed ion current, y-axis) versus time (x-axis), of *M. maripaludis *proteolytic fragments.

**Figure 2:**
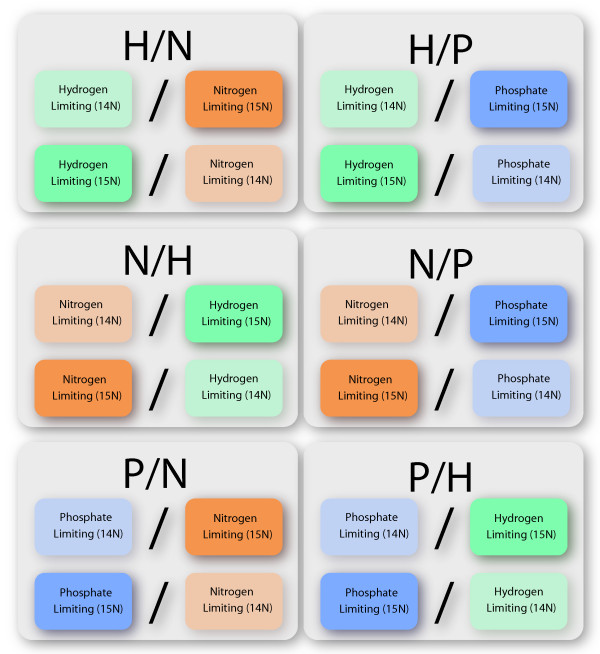
**Experimental design, computational**. The effect of each nutrient limitation was assessed by comparing its proteome to that from the two other nutrient limitations, thus providing two control conditions for each condition under study, green, H_2_-limitation; orange, nitrogen limitation; blue, phosphate limitation; light colors, light isotope (^14^N); dark colors, heavy isotope (^15^N).

Diagrams are provided that show the experimental design for sample handling and mass spectrometry analysis (Figure 1) and nutrient limitation comparisons (Figure 2). To assess the effect of each nutrient limitation, the proteome from that nutrient limitation was directly compared to the proteome from the two other nutrient limitations. For example, the effect of H_2 _limitation was determined from the comparison of H_2_-limited samples (H) to nitrogen-limited samples (N) and phosphate-limited samples (P), yielding H/N ratios and H/P ratios respectively. Similarly, the effect of nitrogen limitation was determined from N/H and N/P ratios, and of phosphate limitation from P/H and P/N ratios. This approach avoided comparison of a nutrient-limited culture to a non-nutrient-limited culture, which would introduce complications arising from variations in growth rate or cell density. Each comparison was conducted by mixing a ^14^N-labeled (natural abundance) sample with a ^15^N-labeled sample after digestion into tryptic fragments but prior to proteomic analysis (Figure 1). As a result of this approach, each nutrient limitation was assessed in a total of four comparisons, using two biological replicates with "flipped" metabolic labels for each nutrient limitation (Figure 2). Proteomics were conducted by 2-D capillary HPLC coupled with tandem mass spectrometry as before [8], with modifications as noted in Methods. Extensive proteome pre-fractionation by HPLC prior to 2-D capillary HPLC as described previously [8] and the modest size of the *M. maripaludis *proteome led to greater sampling depth and proteome coverage (91% of the annotated ORFs were observed experimentally) than is typical for studies of this type [10], essentially saturating each sample in terms of protein identifications. Further repeated replicates would not have led to any significant increase in identifications at the protein level, although a few additional peptides might potentially have been matched with the database. The average number of unique peptide sequences assigned to each detected protein-encoding ORF was 10. The average number of redundant heavy/light and light/heavy peptide pairs recovered for each detected ORF in each comparison of two nutrient limitation states, for purposes of abundance ratio generation, was 198. (Here the term "redundant" refers to measurements that include repeated sampling of the same peptide pair where each observed pair is an estimator of the relative change in protein abundance as in our previous work [8,10].) However, such statistical power is a mixed blessing in that one must then distinguish between real regulatory trends and minor random changes in the system. With so many redundant measurements, it becomes possible to detect very small abundance changes, of magnitude 10% or less, that may or may not have biological meaning [10]. Biological relevance was inferred in part by looking at the consistency of change observed across nutrient limitation comparisons and biological replicates (isotopic flips), as well as the magnitude of the *q*-values for each abundance ratio and the criteria given below.

All ratios and statistical values are provided in Additional file 1. Protein abundance was considered to be affected by a particular nutrient limitation only if a significant difference (log_2 _ratio ≠ 0, *q*-value ≤ 0.01) was seen in all four comparisons described above, except in a few cases where manual inspection of the data suggested that one of the four determinations was an outlier, in which case it was disregarded. qRT-PCR was used to assess mRNA abundance ratios for selected ORFs. These measurements confirmed the proteomic trends in each case tested, and also contributed data supporting the concept that proteomic abundance ratios generated using shotgun methods are compressed [8,10], that is, they tend to underestimate the magnitude of the ratios, especially for highly expressed proteins or high ratios as shown in Tables 1 and 2 and discussed below. The observed compression is consistent with the dynamic range limitations associated with both shotgun proteomics (~10^2 ^to ~10^3^) and mRNA microarray analysis, relative to qRT-PCR [10].

**Table 1:** Selected proteins with altered abundance under H_2 _limitation.

ORF #	Function	Average log_2 _ratio^a^
	Methanogenesis	
MMP0820	FrcA, coenzyme F_420_-reducing hydrogenase	1.30 ± 0.56
MMP1382	FruA, coenzyme F_420_-reducing hydrogenase	0.77 ± 0.16
MMP1384	FruG, coenzyme F_420_-reducing hydrogenase	0.88
MMP1385	FruB, coenzyme F_420_-reducing hydrogenase	0.90
MMP1460	EhaM, energy-conserving hydrogenase A	0.56 ± 0.08
MMP1463	EhaP, energy-conserving hydrogenase A polyferredoxin subunit	0.64 ± 0.27
MMP0058	Mer, methylenetetrahydromethanopterin reductase	0.58
MMP1245	FwdF, formylmethanofuran dehydrogenase	0.20
MMP1247	FwdD, formylmethanofuran dehydrogenase	0.23
MMP1248	FwdA, formylmethanofuran dehydrogenase	0.27
MMP1249	FwdC, formylmethanofuran dehydrogenase	0.28 ± 0.07
MMP1697	HdrA, heterodisulfide reductase	0.35 ± 0.18
MMP1696	VhuD, F_420 _non-reducing hydrogenase	0.35 ± 0.11
MMP1695	VhuG, F_420 _non-reducing hydrogenase	0.34
MMP1694	VhuA, F_420 _non-reducing hydrogenase	0.29
MMP0372	Mtd, F_420_-dependent methylenetetrahydromethanopterin dehydrogenase	0.35 ± 0.10 (0.89)^b^
MMP1054	HdrC2, heterodisulfide reductase	0.33
MMP1053	HdrB2, heterodisulfide reductase	0.33 ± 0.11
MMP1563	MtrB, methyltransferase	0.27 ± 0.16
MMP1564	MtrA, methyltransferase	0.09
MMP0127	Hmd, H_2_-dependent methylenetetrahydromethanopterin dehydrogenase	-2.08 (-3.57)^b^
MMP0125	Hypothetical protein	-1.19
MMP0875	S-layer protein	-1.25
MMP1176	Putative iron transporter subunit	-0.83
MMP1206	GlnA, glutamine synthetase	-0.35

^a^Average of four log_2 _ratios: ^15^N-labeled H_2_-limited compared with ^14^N-labeled nitrogen-limited, ^14^N-labeled H_2_-limited compared with ^15^N-labeled nitrogen-limited, ^15^N-labeled H_2_-limited compared with ^14^N-labeled phosphate-limited, and ^14^N-labeled H_2_-limited compared with ^15^N-labeled phosphate limited. Standard deviations are given. Where protein abundance was affected by a second nutrient limitation (other than H_2_), the average is from two ratios only, each H_2_-limited compared with the non-affecting nutrient limitation.

^b^Values in parentheses represent measurements of mRNA by qRT-PCR.

**Table 2:** Selected proteins with altered abundance under nitrogen limitation.

ORF #	Function	Average log_2 _ratio^a^
	Nitrogen fixation	
MMP0853	NifH, nitrogenase reductase	2.29 ± 0.16
MMP0854	NifI_1_	1.68 ± 0.57
MMP0855	NifI_2_	2.10 ± 0.23
MMP0856	NifD, nitrogenase	2.45 ± 0.15
MMP0857	NifK, nitrogenase	2.03 ± 0.22 (7.09)^b^
MMP0858	NifE	1.85 ± 0.42
MMP0859	NifN	1.65 ± 0.30
MMP0860	NifX	3.13 ± 0.60
MMP0446	NifX-NifB superfamily	1.05 ± 0.40
	Ammonia transport and regulation	
MMP0064	GlnK_1_	1.30
MMP0065	AmtB_1_	2.81 ± 0.31
MMP0066	GlnB	0.45 ± 0.41
MMP0067	GlnK_2_	1.59 ± 0.48
	Ammonia assimilation	
MMP1206	GlnA, glutamine synthetase	1.23
	Molybdate transport	
MMP0205	ModA, molybdate binding protein	2.11 ± 0.47
MMP0507	ModA, molybdate binding protein	2.24 ± 0.50
MMP0516	ModD, molybdate transporter subunit	0.73 ± 0.23

^a^Average of four log_2 _ratios: ^14^N-labeled nitrogen-limited compared with ^15^N-labeled H_2_-limited, ^15^N-labeled nitrogen-limited compared with ^14^N-labeled H_2_-limited, ^15^N-labeled nitrogen-limited compared with ^14^N-labeled phosphate-limited, and ^14^N-labeled nitrogen-limited compared with ^15^N-labeled phosphate limited. Standard deviations are given. Where protein abundance was affected by a second nutrient limitation, average is from two ratios only, each nitrogen-limited compared with the non-affecting nutrient limitation.

^b^Value in parentheses represents measurements of mRNA by qRT-PCR.

### H_2 _limitation

The abundance of 141 proteins (8% of the 1722 annotated ORFs) was significantly affected by H_2_-limitation; 59 had increased abundance and 82 decreased. H/N and H/P ratios and their averages are shown in Additional file 2. The functional category of proteins that most frequently increased was methanogenesis (Table 1). In a previous study at the transcriptome level [5], only a subset of the mRNAs encoding the proteins of methanogenesis was seen to increase significantly; these included the F_420_-reducing hydrogenase (*fru*), methylenetetrahydromethanopterin reductase (*mer*), and methylenetetrahydromethanopterin dehydrogenase (*mtd*), all encoding enzymes that reduce or oxidize coenzyme F_420_. In contrast, in the current study of the proteome, many enzymes in methanogenesis that do not metabolize F_420 _increased as well. Another difference between the results of the previous transcriptome study and the current proteomics study was in the magnitude of the increase for the F_420_-metabolizing enzymes; whereas these mRNAs were previously seen to increase markedly (4–22 fold), the magnitude of change in protein abundance in the current study was at most 2.5-fold. The lower magnitude of change in the current study held at the mRNA level, since qRT-PCR of *mtd *revealed an average log_2 _ratio of only 0.89 (1.9-fold), compared to 4.3 (19.7-fold) in the previous study.

There are several possible reasons why the current study reflects more widespread but less marked changes than the earlier study of the transcriptome. First, our measurement of abundance changes and the significance of those changes have different limitations for the transcriptome and the proteome. Much of the proteome was very heavily sampled in this study, so statistically significant differences are more easily discerned as discussed above. Second, even if the transcriptome study were statistically robust, effects on protein abundance could occur at a post-mRNA level. It should be noted that these first two explanations may apply to the non-F_420_-metabolizing enzymes, but for the F_420_-metabolizing enzymes it is insufficient, based on our qRT-PCR measurements of *mtd*. Third, a caveat to the comparison of the two studies is that growth conditions were different, since the previous study was conducted with a richer medium and at a higher growth rate than the current study. Finally, it should be noted that the strain used in the current study differs from the strain used previously. Mm900, the strain used in the current study, contains a deletion of the *hpt *gene encoding hypoxanthine phosphoribosyltransferase [11], while S52, the strain used in the previous study, is a leucine auxotroph containing a deletion of the *leuA *gene [9]. While the known differences lie outside of hydrogen metabolism or methanogenesis, it is conceivable that there were unknown consequences for methanogenesis. In any case, the results of the current study show that effects of H_2_-limitation occur widely for proteins of methanogenesis. The overall increase in methanogenic proteins with H_2 _limitation likely reflects a regulatory response that maintains flux through the methanogenic pathway when the electron donating substrate is limiting.

One protein decreased strikingly with H_2_-limitation, the H_2_-dependent methylenetetrahydromethanopterin dehydrogenase, Hmd (Table 1). The previous study of the transcriptome [5] indicated an increase in *hmd *mRNA with faster growth, but no change with H_2_-limitation. The discrepancy could be explained by any of the factors discussed above. In any case, the results indicate that Hmd has a decreased role under H_2_-limitation. In hydrogenotrophic methanogens, Hmd catalyses the reduction of methenyltetrahydromethanopterin to methylenetetrahydromethanopterin, using H_2 _directly as electron donor. As such, Hmd provides an alternative to F_420_-reducing hydrogenase (Fru or Frc in *M. maripaludis*) and F_420_-dependent methylenetetrahydromethanopterin dehydrogenase (Mtd) working together: Fru or Frc reduces F_420 _using H_2_, and Mtd reduces methenyltetrahydromethanopterin to methylenetetrahydromethanopterin using reduced F_420_. Hmd is an unusual [Fe] hydrogenase that has a lower affinity for H_2 _than the F_420_-reducing hydrogenases [12,13], and could be preferred when H_2 _is in excess, while Fru or Frc with Mtd could be preferred when H_2 _is limiting. Other proteins that decreased were a hypothetical protein (encoded in a putative operon with Hmd), a putative iron transporter subunit, glutamine synthetase, and an S-layer protein (MMP0875). An additional S-layer protein (MMP0383) was not significantly affected by any nutrient limitation.

### Nitrogen limitation

The abundance of 106 proteins was significantly affected by nitrogen limitation; 79 had increased abundance and 27 decreased. N/H and N/P ratios and their averages are shown in Additional file 3. Of the 79 proteins with increased abundance, 13 have known functions in nitrogen assimilation (Table 2). These are the nitrogen fixation (Nif) proteins, glutamine synthetase (GlnA) which assimilates ammonia, ammonia transporters (Amt), and nitrogen sensor/regulators (GlnK).

Since the Nif proteins showed a consistent and relatively marked increase in abundance, the mRNA encoding one (*nifK*) was selected for qRT-PCR to determine whether the effect occurred with similar magnitude at the transcriptome level. The magnitude was much greater, with an average log_2 _ratio of 7.09 (136-fold) for the mRNA compared to 2.03 (4.1-fold) for the protein. Previous measurements of *nif *transcription using *lacZ *fusions also showed a greater magnitude of regulation (5–100 fold, [14,15]). The results suggest that for proteins that are present at high levels under derepressed conditions, the proteomic ratios may be compressed as noted above. In nitrogen-fixing organisms in general, Nif proteins are present at high levels under nitrogen-fixing conditions when fixed nitrogen species are not available or are present at limiting amounts, but are absent when available fixed nitrogen is in excess.

Other proteins were not previously predicted to function in nitrogen assimilation, yet increased in abundance with nitrogen limitation (Table 2). Three such proteins were predicted subunits of three molybdate transporters, and their response to nitrogen limitation suggests that they function to transport molybdate for conversion into the iron-molybdenum cofactor (FeMoCo) of nitrogenase. A protein belonging to the NifB-NifX family of FeMoCo synthesis proteins also increased.

Surprisingly, several proteins that play central roles in carbon assimilation also increased: subunits of pyruvate oxidoreductase and oxoisovalerate oxidoreductase, as well as acetyl-CoA synthetase (AMP-forming). In hydrogenotrophic methanogens, pyruvate oxidoreductase and oxoisovalerate oxidoreductase each reductively assimilates CO_2_. In addition, ATPase increased moderately (Additional file 3). Proteins that decreased with nitrogen limitation included flagellins, chemotaxis proteins, certain proteins of methanogenesis, and HmdII, a homolog of the H_2_-dependent methylenetetrahydromethanopterin dehydrogenase Hmd. HmdII is not known to have the catalytic activity of Hmd and its function is unknown.

A known transcriptional nitrogen regulator, NrpR, binds to operators with consensus sequence GGAAN_6_TTCC [3,4]. The intergenic regions in *M. maripaludis *that contain this sequence are upstream of the following genes: the *nif *operon, the *glnK*-*amtB *operon, *glnA*, two of the three molybdate transporter operons (MMP0205–0207 and MMP0504–0507), and a gene encoding a Na^+^-alanine symporter (MMP1511). (The Na^+^-alanine symporter may function in nitrogen assimilation since alanine is a nitrogen source for *M. maripaludis*, [11].) Data presented above suggest for all of these genes except the Na^+^-alanine symporter that nitrogen regulation indeed occurs. Furthermore, NrpR-dependent regulation of *nif *and *glnA *has been documented previously [3,4,16]. Since the proteomics data for the Na^+^-alanine symporter was inconclusive, we tested for nitrogen regulation by growing batch cultures on the preferred, intermediate, and non-preferred nitrogen sources ammonia, L-alanine, and N_2_, using a promoter-*lacZ *fusion. β-galactosidase activities were 1060, 2147, and 3122 (standard deviations 21, 193, and 178) respectively, indicating that the gene for the Na^+^-alanine symporter is also regulated by nitrogen. Hence, the following genes are likely regulated directly by NrpR: *nif *and *glnA *as documented previously, the *glnK*-*amtB *operon, the two molybdate transporter operons MMP0205–0207 and MMP0504–0507, and the Na^+^-alanine symporter gene. The remaining proteins affected by nitrogen limitation, including the NifB-NifX homolog and the third molybdate transporter MMP0514–0516, may respond indirectly to NrpR or may be influenced by different mechanisms.

### Phosphate limitation

The abundance of 163 proteins was significantly affected by phosphate limitation; 80 proteins increased and 83 decreased. P/H and P/N ratios and their averages are shown in Additional file 4. The proteins that increased the most markedly were those of a phosphate ABC transporter (Table 3). Homologous phosphate transporters are present in a wide variety of Bacteria, where they are similarly affected by phosphate limitation [17]. Also affected were a homolog of a phosphate transport regulator (PhoU) encoded adjacent to another set of ABC transporter subunits, and a putative Na^+^-phosphate cotransporter. All of these were also affected by phosphate limitation at the mRNA level, with the phosphate ABC transporter (MMP1095–1099) the most markedly regulated [6]. These results suggest that *M. maripaludis *has three different phosphate transporters, all of which are regulated by phosphate conditions. Proteins that decreased with phosphate limitation included the CO_2_-assimilating carbon monoxide dehydrogenase/acetylCoA synthase, acetyl-CoA synthetase (AMP-forming), and certain proteins of methanogenesis (Additional file 4).

**Table 3:** Selected proteins with altered abundance under phosphate limitation.

ORF #	Function	Average log_2 _ratio^a^
	Phosphate transport	
MMP1095	Phosphate binding protein	2.15 ± 0.30
MMP1096	Phosphate transporter subunit	2.12 ± 0.47
MMP1098	ATP binding protein	2.16 ± 0.18
MMP1099	PhoU, regulator	1.16 ± 0.29
MMP1199	PhoU homolog	1.26 ± 0.16
MMP0666	Na^+^/P_i _cotransporter	1.16 ± 0.32

^a^Average of four log_2 _ratios: ^14^N-labeled phosphate-limited compared with ^15^N-labeled H_2_-limited, ^15^N-labeled phosphate-limited compared with ^14^N-labeled H_2_-limited, ^14^N-labeled phosphate-limited compared with ^15^N-labeled nitrogen-limited, and ^15^N-labeled phosphate-limited compared with ^14^N-labeled nitrogen limited. Standard deviations are given.

### Proteins affected by multiple factors

Several proteins were affected by two nutrient limitations (Table 4). A variety were negatively affected by H_2_-limitation and positively affected by nitrogen limitation. Others (proteins encoded in an operon involved in nickel transport and coenzyme M biosynthesis) were negatively affected by H_2_-limitation and positively affected by phosphate limitation. AMP-forming acetylCoA synthetase was affected positively by nitrogen limitation and negatively by phosphate limitation. Flagellins were affected negatively by nitrogen limitation and positively by phosphate limitation. A study in *Methanocaldococcus jannaschii *observed a different effect, where H_2 _limitation resulted in an increase in flagella [18]. That study is not easily compared to the present one, since batch culture was used. However, it is possible that motility and chemotaxis in the two organisms have evolved to increase access to different vital nutrients. Another factor may lie in the ability of *M. maripaludis *but not *M. jannaschii *to fix nitrogen. Nitrogen fixation is an energy-demanding process and *M. maripaludis *under nitrogen fixing conditions may decrease other energy-demanding processes such as motility in order to conserve energy.

**Table 4:** Selected proteins with abundance affected by more than one nutrient limitation.

ORF #	Function	Average log_2 _ratios^a^		
		H_2 _limitation	Nitrogen limitation	Phosphate limitation
MMP0127	Hmd	-2.08	0.68	
MMP0125	Hypothetical protein	-1.19	0.13	
MMP0875	S-layer protein	-1.25	0.76	
MMP1176	Putative iron transporter subunit	-0.83	0.63	
MMP0164	CbiX, cobaltochelatase	-0.59	0.31	
MMP0271	putative nickel transporter	-0.89		0.70
MMP0272	putative nickel transporter	-0.46		0.84
MMP0273	ComA, coenzyme M biosynthesis	-0.58		0.73
MMP0148	acetylCoA synthase, AMP-forming		0.23	-0.98
MMP1666	FlaB1, flagellin precursor		-1.13	0.46
MMP1668	FlaB3, flagellin		-1.04	0.46

^a^Each average log_2 _ratio is derived as described in Tables 1, 2, and 3, and is from the ratios of the nutrient in question with the non-affecting nutrient limitation.

## Conclusion

From this study we have gained new insights into the response of *M. maripaludis *to nutrient limitations. H_2 _limitation affected the proteins of methanogenesis more widely than we had previously appreciated. Many proteins of methanogenesis increased in abundance, in an apparent regulatory response to maintain flux through the methanogenic pathway when H_2 _is limiting. In contrast, the H_2_-dependent methylenetetrahydromethanopterin dehydrogenase (Hmd) decreased. Under H_2_-limitation the function of Hmd may be replaced with the F_420_-dependent methylenetetrahydromethanopterin dehydrogenase (Mtd) together with F_420_-reducing hydrogenase (Frc or Fru).

Many proteins that increased with nitrogen limitation have known functions in nitrogen assimilation and have similarly regulated counterparts in Bacteria and other Archaea [19,20]. Other proteins that increased apparently function in nitrogenase FeMoCo synthesis or to import molybdate for FeMoCo, or to import alanine when used as a nitrogen source. The results help to identify the regulon that is directly regulated by the nitrogen repressor NrpR.

The response to phosphate limitation supports the hypothesis that *M. maripaludis *has three alternative phosphate transporters, all of which increased under phosphate limitation.

## Methods

### Culture conditions

*Methanococcus maripaludis *strain Mm900 [11] was grown in chemostats as described [9], with the following modifications. Amino acid stocks were omitted from the medium, resulting in a defined medium that contained acetate, vitamins, and cysteine as the sole organic constituents. NH_4_Cl was added to the medium after autoclaving from a sterile anaerobic stock. Ar replaced N_2 _in the gas mixture. For growth of nitrogen-limited cultures, NH_4 _^+ ^was decreased to 3 mM in the medium that was pumped into the chemostats, and for growth of phosphate-limited cultures, PO_4 _^2- ^was decreased to 0.15 mM (for sample 31) or 0.13 mM (for sample 82). For growth of H_2_-limited cultures, H_2 _was decreased to 20 ml/min and the difference made up with Ar. The dilution rate in every case was 0.083 h^-1^, and the OD_660 _at harvesting was between 0.62 and 0.71. Two cultures were obtained for each nutrient limitation, one grown with ^14^NH_4 _^+ ^(natural abundance) and the other with ^15^NH_4 _^+ ^supplied as ^15^NH_4_Cl. Sample collection from the chemostats for proteomics was as described [5].

### Proteomics

Proteomic analyses were conducted as described [8], with the primary exception that a Thermo LTQ linear ion trap mass spectrometer (Thermo-Fisher, San Jose, CA) has since replaced the LCQ Classic mass spectrometer for all work reported here. Details of the proteome extraction, trypsin digestion, solution volumes, off-line HPLC fractionation and 2-D capillary HPLC/tandem mass spectrometry, AKA MudPIT [21], Sequest database searching [22], DTASelect 1.9 *in silico *mapping of peptides to *M. maripaludis *protein-encoding ORFs [23], software and database release dates and versions were as described. Briefly, protein was extracted from each of the six cultures depicted in Figure 1. The six protein extracts were digested with trypsin and then combined pair wise as shown in Figure 1, such that equal amounts of heavy (^15^N) and light (^14^N) total protein were used for each condition being compared, as determined by Bradford assay [24,25]. Each of the six combined heavy/light proteolysates shown in Figure 1 were pre-fractionated and analyzed twice by 2-D capillary HPLC/tandem mass spectrometry. The data from the two technical replicates were pooled, yielding a single dataset for each heavy/light mixture. These mass spectrometry datasets (see Additional data files) were labeled in the Hackett Lab archive as AH30-31-104 (^14^N phosphate, ^15^N ammonia), AH30-31-49 (^14^N phosphate, ^15^N hydrogen), AH30-49-98 (^15^N hydrogen, ^14^N ammonia), AH30-54-104 (^14^N hydrogen, ^15^N ammonia) AH30-82-54 (^15^N phosphate, ^14^N hydrogen) and AH30-82-98 (^15^N phosphate, ^14^N ammonia). To ensure that equimolar amounts of total protein were being compared, the Bradford assay results were confirmed by inspecting the calculated abundance ratio frequency distribution histograms for zero centering (log_2 _scale) and making slight adjustments in the ratios where necessary [8]. In no case did the normalization of the ratios exceed a 5% change in the total signal observed in either channel (^14^N or ^15^N). Raw data from the six heavy/light mixtures (Figure 1) were processed as described previously, except as noted below, to yield abundance ratios reported in Additional file 1. Figure 2 illustrates the use of the abundance ratios derived from the six unique mixtures (Figure 1) of isotopic flips to calculate the total of 12 two-condition comparisons with four abundance ratios for each of the three limiting nutrient conditions, as reported in Additional files 2, 3, 4 for proteins showing significant abundance change. Statistical analysis differed from that reported previously [8] in that a *q*-value [26,27] cut-off of 0.01 was used for all significance testing for abundance change between paired conditions, rather than *p*-values. The *q*-value is based on the concept of FDR (false discovery rate) and contains an explicit correction for multiple hypothesis testing that is lacking in an uncorrected *p*-value calculation [26]. At the level of qualitative peptide identifications, the estimated FDRs for the work reported here were ~3%, based on matches with reversed protein sequences in the decoy portion of the database [28,29]. Along with a minimum requirement of three unique peptide sequences required for each identification, this estimate suggests a low number of false positive protein level identifications. The composition, release dates, and other details of the FASTA database were the same as those reported previously [8], with the exception that the database has been approximately doubled in size to 40 Mbytes by addition of reversed sequences to the forward protein sequences for *M. maripaludis *(Genbank™ Accession BX950229) and addition of about 25% of the human subset of the nrdb [30]. For purposes of validating protein derived abundance ratios, qRT-PCR was conducted as described [8].

### Alanine transporter-*lacZ *fusion

The promoter of the Na^+^-alanine symporter (MMP1511) gene was PCR-amplified from *M. maripaludis *S2 [31] genomic DNA using primers 5'AAACTAGTAATCAAGTATTTAAATCCGTTAC3' (forward) and 5' ACCATGCATCCACTCCAAATTTTTTTGG (reverse). Herculase^® ^(Stratagene) was used and conditions were 94°C for 2 min; 30 cycles of 94°C for 30 sec, 51°C for 30 sec, and 68°C for 30 sec; and a final extension of 68°C for 10 min. Product was digested with *Spe*I and *Nsi*I and cloned into pWLG40+lacZ to yield pWLG40agcsB2-1. Plasmid DNA was transformed [32] into Mm900 to give Mm1086. Growth of Mm1086 and β-galactosidase assay were as described [14]. Measurements were taken from triplicate cultures.

## Abbreviations

2-D: two dimensional; Amt: ammonia transporter; F_420_: coenzyme F_420_; FDR: false discovery rate; FeMoCo: iron-molybdenum cofactor; Fru: F_420_-reducing hydrogenase (selenocysteine-containing); GlnA: glutamine synthetase; GlnK: nitrogen sensor/regulator; H: H_2_-limited; Hmd: H_2_-dependent methylenetetrahydromethanopterin dehydrogenase; HPLC: high performance liquid chromatography; Mer: methylenetetrahydromethanopterin reductase; Mtd: methylenetetrahydromethanopterin dehydrogenase (F_420_-dependent); N: nitrogen-limited; Nif: nitrogen fixation; NrpR: nitrogen regulatory protein; P: phosphate-limited; qRT-PCR: quantitative real-time reverse transcription-PCR.

## Authors' contributions

QX and TW performed protein biochemistry, 2-D capillary HPLC separations, mass spectrometry and data analysis. ELH performed data analysis and bioinformatics. TJL assayed expression of the Na^+^-alanine symporter gene. MH and JAL supervised the research. JAL wrote the manuscript.

## Supplementary Material

Additional file 1 **Complete list of protein abundance ratios, *p*-values, and *q*-values**. Complete data set, with log_2 _ratios, *p*-values, *q*-values, and abundance trends (up, down, or no significant difference). Click here for file

Additional file 2 **Proteins with altered abundance under H_2 _limitation**. Log_2 _ratios for proteins with altered abundance under H_2 _limitation. Click here for file

Additional file 3 **Proteins with altered abundance under nitrogen limitation**. Log_2 _ratios for proteins with altered abundance under nitrogen limitation. Click here for file

Additional file 4 **Proteins with altered abundance under phosphate limitation**. Log2 ratios for proteins with altered abundance under phosphate limitation. Summary tables of individual proteomic results at the peptide level, in the form of *DTASelect *Ver. 1.9 filter files [23], are posted at http://depts.washington.edu/mhlab/Mm900nutrientlimitation. Log in with user name **MMP **and password **threebugs**. These files are organized by the archive names given under Materials and Methods and contain Sequest [22] scores for individual peptide mass spectra, search parameters and other detailed information that can be used to assess data quality at multiple levels, i.e. peptides, proteins and individual CID (MS^2^) mass spectra. The *sequest.params *file for each analysis is also posted. Researchers interested in the raw data (*.RAW files) should contact mhackett@u.washington.edu. Click here for file
